# Peptide 19-2.5 Inhibits Heparan Sulfate-Triggered Inflammation in Murine Cardiomyocytes Stimulated with Human Sepsis Serum

**DOI:** 10.1371/journal.pone.0127584

**Published:** 2015-05-29

**Authors:** Lukas Martin, Susanne Schmitz, Rebecca De Santis, Sabine Doemming, Hajo Haase, Janine Hoeger, Lena Heinbockel, Klaus Brandenburg, Gernot Marx, Tobias Schuerholz

**Affiliations:** 1 Department of Intensive Care and Intermediate Care, University Hospital, Aachen, Germany; 2 Department of Food Chemistry and Toxicology, Berlin Institute of Technology, Berlin, Germany; 3 Institute of Immunology, Medical Faculty, RWTH Aachen University, Aachen, Germany; 4 Division of Biophysics, Forschungszentrum, Borstel, Germany; Klinikum rechts der Isar—Technical University Munich—TUM, GERMANY

## Abstract

Myocardial dysfunction in sepsis has been linked to inflammation caused by pathogen-associated molecular patterns (PAMPs) as well as by host danger-associated molecular patterns (DAMPs). These include soluble heparan sulfate (HS), which triggers the devastating consequences of the pro-inflammatory cascades in severe sepsis and septic shock. Thus, there is increasing interest in the development of anti-infective agents, with effectiveness against both PAMPs and DAMPs. We hypothesized that a synthetic antimicrobial peptide (peptide 19-2.5) inhibits inflammatory response in murine cardiomyocytes (HL-1 cells) stimulated with PAMPs, DAMPs or serum from patients with septic shock by reduction and/or neutralization of soluble HS. In the current study, our data indicate that the treatment with peptide 19-2.5 decreases the inflammatory response in HL-1 cells stimulated with either PAMPs or DAMPs. Furthermore, our work shows that soluble HS in serum from patients with Gram-negative or Gram-positive septic shock induces a strong pro-inflammatory response in HL-1 cells, which can be effectively blocked by peptide 19-2.5. Based on these findings, peptide 19-2.5 is a novel anti-inflammatory agent interacting with *both* PAMPs and DAMPs, suggesting peptide 19-2.5 may have the potential for further development as a broad-spectrum anti-inflammatory agent in sepsis-induced myocardial inflammation and dysfunction.

## Introduction

Sepsis remains one of the most common cause of death in intensive care units worldwide [1]. Thereby septic cardiomyopathy is recognized in at least 50% of patients with septic shock and its presence indicates a worse prognosis [2]. Today, it is known that toll-like receptors (TLRs) on cardiomyocytes initiate a NFκB dependent inflammation during sepsis, which leads to myocardial contractile dysfunction [3]. In recent decades it has become evident that there are two main signaling pathways that induce inflammation in sepsis: one is by pathogen-associated molecular patterns (PAMPs), such as lipopolysaccharide (LPS) or lipopeptide, the other one by host danger-associated molecular patterns (DAMPs), which alert the immune system to tissue damage following both infectious and sterile insults [4]. Here, heparan sulfate proteoglycans play a key role [5,6]. They are localized in the endothelial glycocalyx and consist of a core membrane-anchored protein with attached heparan sulfate (HS) side chains. In the course of inflammation, HS side chains can be rapidly shed from their proteoglycans [7,8]. Once liberated, HS acts as a DAMP and triggers the devastating consequences of the pro-inflammatory cascades in severe sepsis and septic shock [9,10]. Due to this fact, there is increasing interest in the development of new anti-infective agents, with effectiveness against both PAMPs and DAMPs. Naturally occurring antimicrobial peptides are capable of neutralizing microbial immunostimulatory cell wall compounds as well as endogenous DAMPs [11], however their therapeutic application is limited due to intrinsic toxicity [12,13]. Peptide 19–2.5 belongs to the class of newly developed synthetic antimicrobial peptides (SALP = synthetic anti-LPS peptides) which are able to neutralize LPS *in vitro* and *in vivo* [14,15]. Furthermore, peptide 19–2.5 shows anti-inflammatory effects in Gram-positive, polymicrobial or viral infection, suggesting a DAMP-associated, pathogen type-independent mechanism [14–18].

We hypothesized that peptide 19–2.5 attenuates inflammatory response in cardiomyocytes stimulated with PAMPs, DAMPs or serum from patients with Gram-negative or Gram-positive septic shock. Furthermore, we expected that the broad-spectrum anti-inflammatory effect of peptide 19–2.5 is caused by reduction and/or neutralization of circulating soluble HS. Since the myocardium is a tissue with high blood flow during sepsis [19], we used an established cell culture model of murine cardiomyocytes (HL-1 cells). These cells retain adult cardiac morphological and biochemical properties, including TLR-expression and biochemical NFκB dependent response to TLR-ligands [3,20]. We measured NFκB-activity as well as mRNA expression and secreted protein concentrations of tumor necrosis factor-α (TNF-α) and interleukin-6 (IL-6) to investigate the inflammatory response.

Peptide 19–2.5 was shown to decrease the inflammatory response in HL-1 cells stimulated with PAMPs, DAMPs or serum from patients with septic shock. Interestingly, soluble HS in serum from patients with Gram-negative or Gram-positive septic shock induced a strong pro-inflammatory response in HL-1 cells, which could be effectively blocked by peptide 19–2.5. Thus, our study demonstrates that the antimicrobial peptide 19–2.5 has a broad-spectrum anti-inflammatory activity by interacting with *both* PAMPs and DAMPs.

## Materials and Methods

### Cell culture

HL-1 cardiomyocytes were originally purchased from William Claycomb, Louisiana State University (kindly provided from Dr. Andreas Goetzenich, University Hospital Aachen) [20]. The cells were grown on 5 μg/ml fibronectin and 0.02% gelatin. As described before [20], cells were maintained in supplemented Claycomb medium and incubated under an atmosphere of 5% CO_2_ and 95% air at 37°C. Cultures were grown to high density and passaged 1:2 to 1:3 every three to four days when full confluence was reached to ensure the clones retained their differentiated characteristics. The medium was changed every 24 h. Cells were passaged by adding trypsin-EDTA (Sigma-Aldrich, St. Louis, MO, USA) to the culture dishes for 5–10 minutes. Trypsin activity was blocked by the trypsin inhibitor from glycine max (soya bean; Sigma-Aldrich, St. Louis, MO, USA) at a ratio of 10 μl per 1 cm^2^ of cells.

### Transfections, stimulation and luciferase assays

HL-1 cells were plated on 6-well plates 48–72 h before transfection. The transfection complex contained per plate 635 μl of supplemented Claycomb medium, 49.1 μl of FuGENE HD transfection reagent, and 16.3 μg of DNA. The cells were transfected using the *firefly* pGL4.32 [luc2P/NFκB-RE/Hygro] vector and the *Renilla* pGL4.74 [hRluc/TK] vector (all Promega, WI, USA). A total of 15.6 μg of luc2P/NFκB pGL4.32 was used in conjunction with 0.66 μg of Renilla pGL4.74 per plate. The transfection complex was added to the cell culture and incubated for 24 h, followed by additional 24 h incubation in supplemented Claycomb medium. LPS from the rough mutant Ra from Salmonella enterica serovar Minnesota (R60) was extracted as described in [15]. LPS, fibroblast stimulating lipopeptide-1 (FSL-1) (EMC Microcollections, Tübingen, Germany), HS (AMS Biotechnology, Oxon, United Kingdom), serum from patients with Gram-negative or Gram-positive septic shock or HS-free serum was added in different concentrations to the supplemented Claycomb medium 4 h prior to luciferase measurement, in the presence or absence of peptide 19–2.5 (20 μg/ml, sequence GCKKYRRFRWKFKGKFWFWG, molecular weight 2711). In preceding experiments the dose of 20 μg/ml peptide 19–2.5 combined the highest efficiency with the lowest toxicity [15]. NFκB-Luciferase activity was assayed with the Dual-Glo Luciferase system (Promega, Madison, WI, USA) as per the manufacturer's instructions. Firefly luciferase values were normalized to Renilla luciferase values for each set of readings as per the manufacturer's instructions.

### RNA extraction and PCR

Total RNA was prepared using TRIzol reagent (Invitrogen, Carlsbad, CA, USA). For reverse transcription, 2 μg of RNA, random primers and 150 units of M-MLV RT (Promega, Madison, WI, USA) were used. cDNA was analyzed by quantitative real-time PCR performed with Power SYBR Green PCR Master Mix on a StepOnePlus (all life technologies, Carlsbad, CA, USA) using the following primers: TNF-α 5’ *TCCCCAAAGGGATGAGAAG* 3’ (for) and 5’ *GCACCACTAGTTGGTTGTC* 3’ (rev); and IL-6 5’ *GAGGATACCACTCCCAACAGACC* 3’ (for) and 5’ *AAGTGCATCATCGTTGTTCATACA* 3’ (rev). Ribosmal Protein S7 was used as an endogenous normalization control: 5’ *GGTGGTCGGAAAGCTATCA* 3’(for) and 5’ *AAGTCCTCAAGGATGGCGT 3’* (rev). The following conditions were used: initial denaturation for for 3 minutes at 95°C, followed by 40 cycles at 95°C for 30 seconds, 57°C for 30 seconds and 72°C for 30 seconds.

### Determination of secreted cytokines in HL-1 cells

Levels of secreted pro-inflammatory cytokines were measured using ELISA. Therefore cell supernatants were collected after 4h stimulation. The amounts of IL-6 (NOVEX, San Diego, CA) and TNF-α (Invitrogen, Camarillo, CA) were determined according to the manufacturer’s instructions. The absorbance was measured at 450 nm on a microplate reader (Sunrise Tecan, Crailsheim, Germany).

### Study population

We sampled serum and plasma from 18 patients consecutively admitted to the intensive care unit within 24h after presentation with Gram-negative (n = 10) or Gram-positive (n = 8) septic shock, according to the ACCP/SCCM definitions [21]. In all cases, patients had positive blood cultures with either Gram-negative or Gram-positive strains. Furthermore, we collected plasma from healthy human donors (n = 10). To stimulate HL-1 cells we used the sera from 6 of the 18 septic shock patients with either Gram-negative (n = 3) or Gram-positive (n = 3) strain of infection (Table 1).

**Table 1:** Characteristics of serum from septic shock patients used for cell stimulation.

Patient no.	Age	Sex	Infecting organism	IL-6 level (pg/ml)	HS level (μg/ml)	APACHE II score	28-days outcome
**1**	75	Male	E.coli	5206,5	170,0	22	Alive
**2**	76	Male	E.coli	702,9	213,5	30	Alive
**3**	63	Female	Enterobacter aerogenes	546,6	158,1	20	Alive
**4**	82	Female	Staph. aureus (MRSA)	575,5	118,6	29	Dead
**5**	75	Female	Staph. epidermidis	957,1	104,4	25	Dead
**6**	60	Male	Streptococcus anginosus	2156,2	128,3	22	Alive

IL-6 (interleukin-6), HS (heparan sulfate), MRSA (methicillin resistant staphylococcus aureus), APACHE (Acute Physiology And Chronic Health Evaluation)

### Ethics statement

This study and the collection of serum and plasma were approved by the local ethics committee of the University Hospital Aachen (EK_206_09). All patients or their legal representative gave written informed consent before sampling.

### Heparan sulfate ELISA

The amount of HS in serum and plasma was determined using ELISA (AMS Biotechnology, Oxon, United Kingdom) according to the manufacturer’s instructions. The absorbance was measured at 450 nm on a microplate reader (Sunrise Tecan, Crailsheim, Germany).

### Elimination and reconstitution of HS in serum of septic shock patients *in vitro*

HS was eliminated from serum (n = 6) of septic shock patients (SsP) *in vitro* using a biotin-conjugated polyclonal antibody against HS (host: rabbit, clone: PAA565Hu71, USCN Life Science Ltd Co., Wuhan, China) and affinity chromatography (Pierce Streptavidin Agarose Columns, Thermo Scientific Inc., Worcester, MA, USA) according to the manufacturers’ instructions. SsP was incubated with anti-HS antibody (1:200 dilution) for 10min at room temperature and added to the column. The column was placed in a collection tube and centrifuged at 500 x g for 1 minute. A specific ELISA (AMS Biotechnology, Oxon, United Kingdom) was used to test the absence of HS in SsP according to the manufacturer’s instructions. To exclude that other factors are co-eliminated we reconstituted the detected amount of HS with artificial HS (AMS Biotechnology, Oxon, United Kingdom) to each sample (reconstituted serum) and re-performed the measurements.

### Statistical analyses

The PCR-derived data were derived using a relative expression software tool (REST, (http://www.gene-quantification.de/rest.html, rest-mcs-beta-9august 2006) [22]. The expression ratios are calculated on the basis of the mean crossing point (CP) values for reference and target genes. All data are given as mean ± standard deviation (SD). We used a multiple t-test with Holm-Šídák correction when comparing differences between experimental (peptide treatment) and control (untreated cells) groups. We used a 1-way-ANOVA and Tukey´s-Test for multiple comparisons when comparing differences in heparan sulfate levels between healthy volunteers and septic shock patients with Gram-negative or Gram-positive strain of infection. We performed all calculation and figures using GraphPad Prism 6 (GraphPad, San Diego, CA, USA). A p-value of p < 0.05 was considered significant.

## Results

### PAMPs-mediated inflammatory response in cell culture

**Fig 1:**
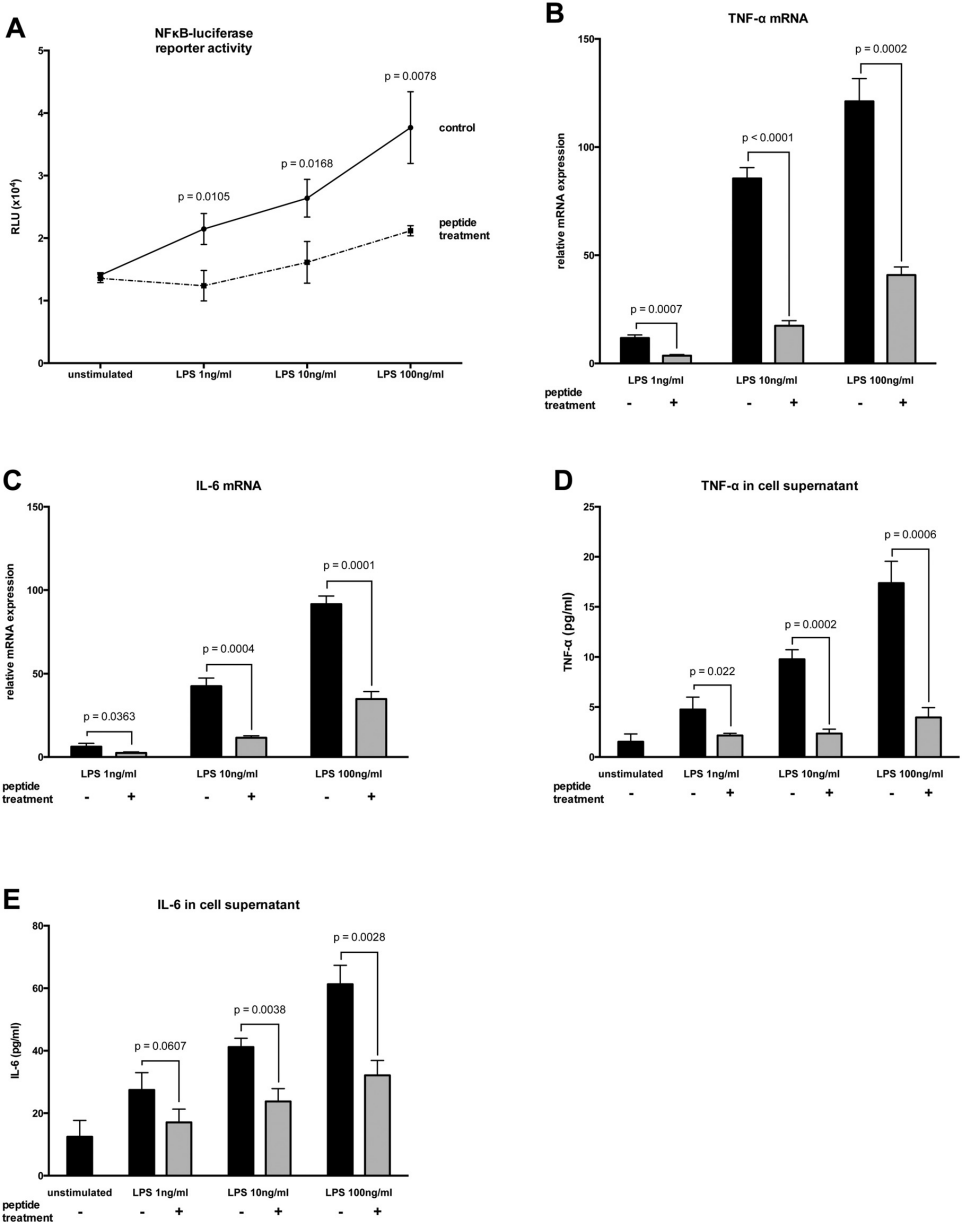
Inflammatory responses in HL-1 cells stimulated with LPS. (A) The cells were stimulated for 4 h with different concentrations of lipopolysaccharide (LPS) and treated with peptide 19–2.5 (20 μg/ml; dashed line) or 0.9% NaCl (control; solid line). The data are expressed as the ratio of firefly to Renilla luciferase activity in relative light units (RLU). (B and C) Following a 4-h stimulation, the induction of mRNAs encoding the pro-inflammatory TNF-α (B) and IL-6 (C) cytokines was monitored by RT-PCR. The inductions shown are normalized to non-stimulated cells. (D and E) Secreted protein concentrations of TNF-α (D) and IL-6 (E) were determined in supernatants using ELISA. Data represent the mean ± SD of triplicate samples, representative of three independent experiments. P values represent statistically significance between untreated (control) and treated cells with peptide 19–2.5 (peptide treatment) using multiple t-test with Holm-Šídák correction.

**Fig 2:**
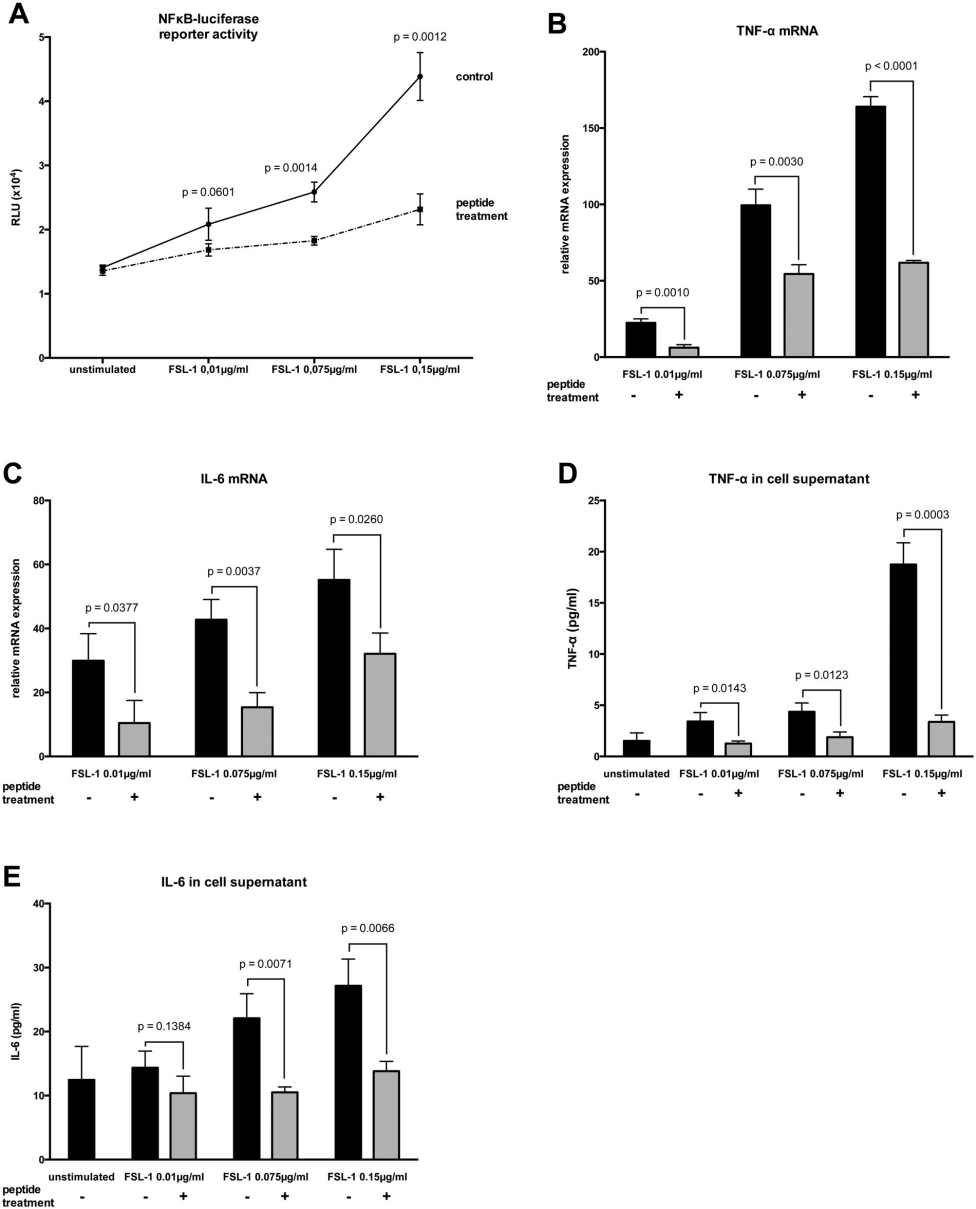
Inflammatory responses in HL-1 cells stimulated with FSL-1. (A) The cells were stimulated for 4 h with different concentrations of fibroblast stimulating lipopeptide-1 (FSL-1) and treated with peptide 19–2.5 (20 μg/ml; dashed line) or 0.9% NaCl (control; solid line). The data are expressed as the ratio of firefly to Renilla luciferase activity in relative light units (RLU). (B and C) Following a 4-h stimulation, the induction of mRNAs encoding the pro-inflammatory TNF-α (B) and IL-6 (C) cytokines was monitored by RT-PCR. The inductions shown are normalized to non-stimulated cells. (D and E) Secreted protein concentrations of TNF-α (D) and IL-6 (E) were determined in supernatants using ELISA. Data represent the mean ± SD of triplicate samples, representative of three independent experiments. P values represent statistically significance between untreated (control) and treated cells with peptide 19–2.5 (peptide treatment) using multiple t-test with Holm-Šídák correction.

Stimulation of HL-1 cells with lipopolysaccharide (LPS) from Gram-negative or fibroblast stimulating lipopeptide-1 (FSL-1) from Gram-positive bacteria resulted in a significant and dose-dependent increase in NFκB-luciferase reporter activity (Figs 1A and 2A) compared to unstimulated cells. Additionally, the mRNA levels (Figs 1B and 1C and 2B and 2C) and secreted protein concentrations (Figs 1D,1E and 2D,2E) of TNF-α and IL-6 were significantly upregulated relative to non-stimulated cells. Treatment with peptide 19–2.5 significantly lowered NFκB-luciferase reporter activity levels (Figs 1A and 2A) and significantly decreased TNF-α and IL-6 mRNA expression (Figs 1B,1C and 2B,2C) and secreted protein concentrations (Figs 1D,1E and 2D,2E) compared to untreated cells.

### HS-mediated inflammatory response in cell culture

**Fig 3:**
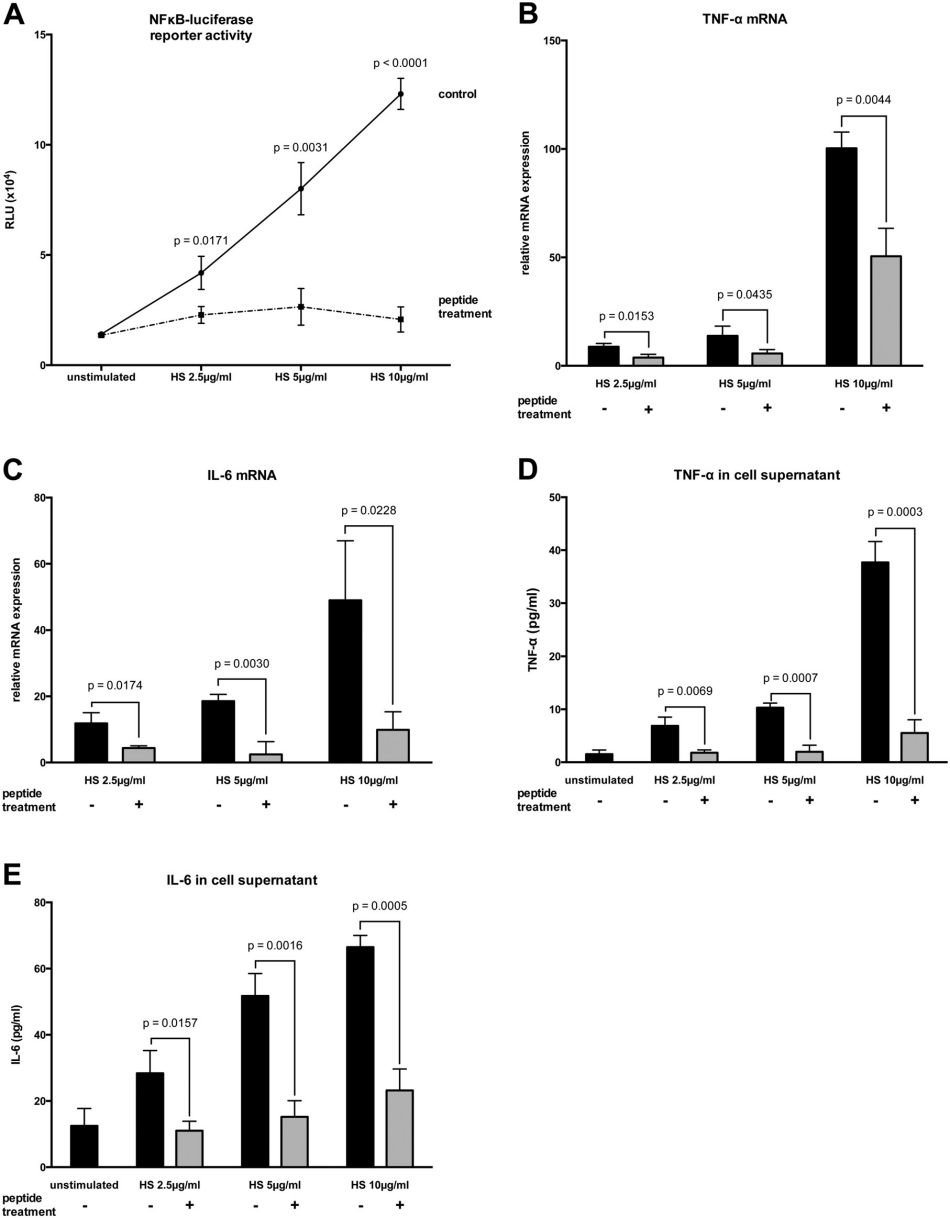
HS-mediated inflammatory response in HL-1 cells. (A) The cells were stimulated for 4 h with different concentrations of heparan sulfate (HS) and treated with peptide 19–2.5 (20 μg/ml; dashed line) or 0.9% NaCl (control; solid line). The data are expressed as the ratio of firefly to Renilla luciferase activity in relative light units (RLU). (B and C) HL-1 cells were stimulated as described for A. Following a 4-h stimulation, the induction of mRNAs encoding the pro-inflammatory TNF-α (B) and IL-6 (C) cytokines were monitored by RT-PCR. The inductions shown are normalized to non-stimulated cells. (D and E) Secreted protein concentrations of TNF-α (D) and IL-6 (E) were determined in supernatants using ELISA. Data represent the mean ± SD of triplicate samples, representative of three independent experiments. P values represent statistically significance between untreated (control) and treated cells with peptide 19–2.5 (peptide treatment) using multiple t-test with Holm-Šídák correction.

Stimulation of HL-1 cells with HS resulted in a significant dose-dependent increase in NFκB-luciferase reporter activity compared to non-stimulated cells (Fig 3A). Furthermore, the mRNA levels and secreted protein concentrations of TNF-α and IL-6 were upregulated in HL-1 cells stimulated with HS in a dose-dependent manner relative to non-stimulated cells (Fig 2B–2E). Treatment with peptide 19–2.5 significantly lowered NFκB-luciferase reporter activity levels (Fig 2A) and significantly decreased TNF-α and IL-6 mRNA expression and secreted protein concentrations in HL-1 cells stimulated with HS compared to untreated cells (Fig 2B–2E).

### Inflammatory response in cells stimulated with human septic shock serum

**Fig 4:**
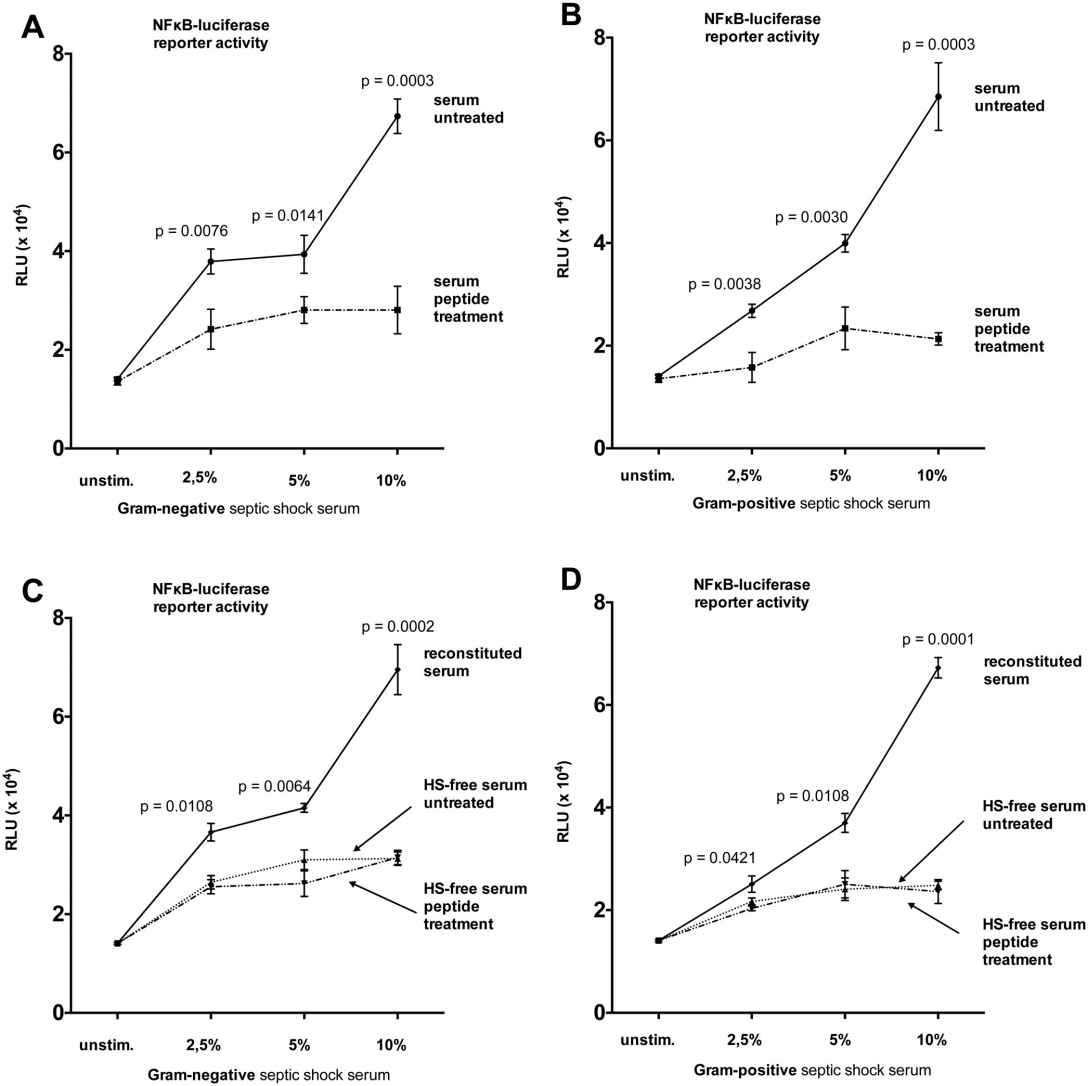
NFκB-luciferase reporter activity in HL-1 cells stimulated with sera from patients with septic shock. HL-1 cells were stimulated with sera from patients with Gram-negative (A) or Gram-positive (B) septic shock and treated with peptide 19–2.5 (20 μg/ml, peptide treatment) or untreated (control). HS has been eliminated from the sera and cells were stimulated with HS-free serum and treated with peptide 19–2.5 (20 μg/ml, peptide treatment) or untreated (HS-free serum) (C and D). The detected amount of HS was reconstituted using artificial HS to each serum sample of HS-free serum and cells were stimulated with reconstituted serum (C and D). The data are expressed as the ratio of firefly to Renilla luciferase activity in relative light units (RLU). Data represent the mean ± SD of triplicate samples, representative of three independent experiments. P values represent statistically significance between serum untreated and serum peptide treatment (A and B) or reconstituted serum and HS-free serum untreated (C and D) using multiple t-test with Holm-Šídák correction.

**Fig 5:**
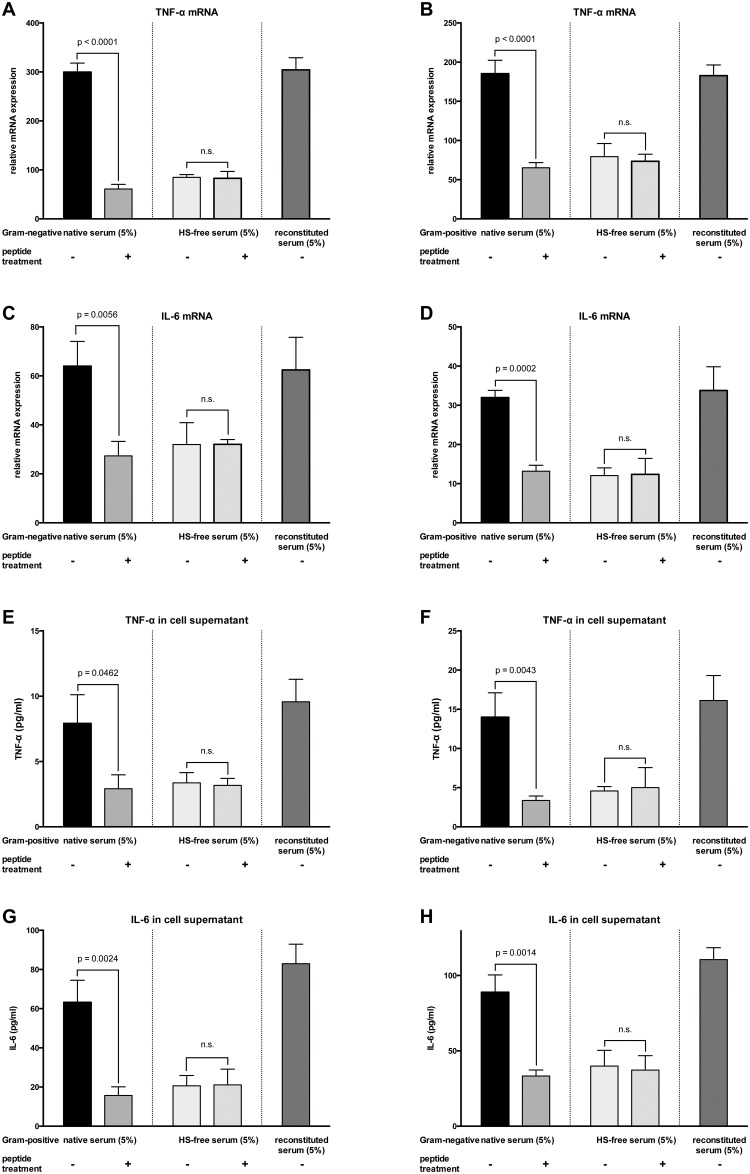
TNF-α and IL-6 mRNA expressions and secreted protein concentrations of HL-1 cells stimulated with sera from patients with septic shock. HL-1 cells were stimulated as described in Fig 4 and induction of mRNAs encoding the pro-inflammatory cytokines TNF-α (A and B) and IL-6 (C and D) were measured. (E and H) Secreted protein concentrations of TNF-α and IL-6 were determined in supernatants using ELISA. Fig 5 shows the data for stimulation with 5% serum, other concentrations see Tables 2 and 3. Data represent the mean ± SD of triplicate samples, representative of three independent experiments. P values represent statistically significance between untreated and treated cells with peptide 19–2.5 using multiple t-test with Holm-Šídák correction.

To investigate whether peptide 19–2.5 reduces inflammation induced by human sepsis serum we tested the inflammatory response of HL-1 cells stimulated with serum from patients with Gram-negative or Gram-positive septic shock at different concentrations. NFκB-luciferase reporter activity significantly increased after stimulation with serum from patients with Gram-negative or Gram-positive septic shock compared to non-stimulated cells (Fig 4A and 4B). The mRNA levels and secreted protein concentrations of TNF-α and IL-6 were significantly upregulated relative to non-stimulated cells (stimulation with 5% serum see left part of Fig 5A–5H, other concentrations see Tables 2 and 3). Treatment with peptide 19–2.5 significantly lowered NFκB-luciferase reporter activity levels (Fig 4A and 4B) and significantly decreased mRNA expression and secreted protein concentrations of TNF-α and IL-6 in HL-1 cells stimulated with serum from septic shock patients compared to untreated cells (Fig 5A–5H, left part and Tables 2 and 3).

**Table 2:** TNF-α mRNA expressions and secreted protein concentrations of HL-1 cells stimulated with sera from patients with septic shock.

			Untreated cells	Peptide treatment	P value
**TNF-α relative mRNA expressions**					
**Gram-negative**	**serum**	2.5%	163.75 ± 6.36	20.09 ± 4.96	< 0.0001
		5%	299.66 ± 18.55	60.99 ± 9.48	< 0.0001
		10%	537.74 ± 89.38	80.38 ± 11.51	0.0009
	**HS-free serum**	2.5%	65.31 ± 8.98	64.74 ± 4.77	0.9264
		5%	84.79 ± 5.68	82.69 ± 14.17	0.8233
		10%	136.08 ± 26.12	130.28 ± 13.23	0.7488
	**reconstituted serum**	2.5%	146.83 ± 11.65	-	-
		5%	304.26 ± 24.81	-	-
		10%	449.26 ± 88.16	-	-
**Gram-positive**	**serum**	2.5%	59.81 ± 7.54	8.71 ± 3.17	0.0008
		5%	185.31 ± 17.14	65.25 ± 6.56	< 0.0001
		10%	255.49 ± 25.96	86.18 ± 9.94	< 0.0001
	**HS-free serum**	2.5%	28.31 ± 11.11	27.48 ± 7.03	0.9332
		5%	79.37 ± 16.79	73.52 ± 8.98	0.5592
		10%	104.87 ± 5.17	108.46 ± 17.10	0.7187
	**reconstituted serum**	2.5%	59.88 ± 3.60	-	-
		5%	182.75 ± 13.64	-	-
		10%	226.96 ± 20.79	-	-
**TNF-α secreted protein concentrations**					
**Gram-negative**	**serum**	2.5%	3.9 ± 1.3	2.8 ± 0.6	0.2542
		5%	16.0 ± 3.1	3.4 ± 0.6	0.0043
		10%	58.2 ± 2.7	14.2 ± 3.9	< 0.0001
	**HS-free serum**	2.5%	3.4 ± 0.6	3.4 ±. 1.1	0.9631
		5%	4.6 ± 0.6	5.0 ± 2.6	0.7899
		10%	11.7 ± 1.3	11.9 ± 3.4	0.9141
	**reconstituted serum**	2.5%	5.8 ± 0.5	-	-
		5%	16.1 ± 3.2	-	-
		10%	64.6 ± 5.2	-	-
**Gram-positive**	**serum**	2.5%	4.1 ± 0.9	2.3 ± 0.2	0.4832
		5%	7.9 ± 2.2	2.9 ± 1.1	0.0462
		10%	37.9 ± 7.1	8.0 ± 1.6	< 0.0001
	**HS-free serum**	2.5%	3.3 ± 0.6	2.6 ± 0.5	0.6160
		5%	3.4 ± 0.8	3.2 ± 0.5	0.8964
		10%	6.6 ± 2.0	7.2 ± 3.6	0.6869
	**reconstituted serum**	2.5%	6.0 ± 1.1	-	-
		5%	9.6 ± 1.7	-	-
		10%	47.2 ± 10.7	-	-

TNF-α (tumor necrosis factor α), HS (heparan sulfate). Data represent the mean ± SD of triplicate samples, representative of three independent experiments. P values represent statistically significance between untreated and treated cells with peptide 19–2.5 using multiple t-test with Holm-Šídák correction.

**Table 3:** IL-6 mRNA expressions and secreted protein concentrations of HL-1 cells stimulated with sera from patients with septic shock.

			Untreated cells	Peptide treatment	P value
**IL-6 relative mRNA expressions**					
**Gram-negative**	**serum**	2.5%	31.08 ± 5.63	12.80 ± 2.52	0.0068
		5%	63.97 ± 10.11	27.35 ± 5.90	0.0056
		10%	192.16 ± 64.17	65.82 ± 12.48	0.0286
	**HS-free serum**	2.5%	12.06 ± 1.96	14.13 ± 3.61	0.4325
		5%	31.97 ± 8.96	32.05 ± 1.96	0.9878
		10%	55.10 ± 5.41	53.48 ± 6.84	0.7641
	**reconstituted serum**	2.5%	28.13 ± 4.58	-	-
		5%	62.46 ± 13.32	-	-
		10%	200.08 ± 26.49	-	-
**Gram-positive**	**serum**	2.5%	34.21 ± 10.60	6.28 ± 3.87	0.0128
		5%	31.97 ± 1.83	13.18 ± 1.51	0.0002
		10%	192.16 ± 64.17	34.46 ± 1.67	0.0131
	**HS-free serum**	2.5%	12.00 ± 4.01	8.85 ± 2.88	0.3325
		5%	12.06 ± 1.96	12.39 ± 4.05	0.9069
		10%	28.12 ± 5.68	26.81 ± 4.05	0.7687
	**reconstituted serum**	2.5%	32.58 ± 6.67	-	-
		5%	33.79 ± 6.03	-	-
		10%	199.73 ± 39.63	-	-
**IL-6 secreted protein concentrations**					
**Gram-negative**	**serum**	2.5%	37.3 ± 3.4	14.0 ± 5.5	0.0034
		5%	88.7 ± 11.5	33.3 ± 4.1	0.0014
		10%	139.9 ± 20.6	35.5 ± 2.5	0.0009
	**HS-free serum**	2.5%	19.3 ± 1.2	20.0 ± 2.5	0.6922
		5%	39.9 ± 10.4	37.2 ± 9.6	0.7601
		10%	54.9 ± 4.5	41.3 ± 2.0	0.0936
	**reconstituted serum**	2.5%	45.2 ± 5.6	-	-
		5%	110.5 ± 7.9	-	-
		10%	140.9 ± 25.6	-	-
**Gram-positive**	**serum**	2.5%	20.2 ± 1.9	13.0 ± 3.8	0.0426
		5%	63.2 ± 11.3	15.7 ± 4.4	0.0024
		10%	94.4 ± 6.5	34.4 ± 5.4	0.0002
	**HS-free serum**	2.5%	13.6 ± 2.5	13.2 ± 2.6	0.8684
		5%	20.6 ± 5.3	21.1 ± 8.1	0.9349
		10%	34.0 ± 6.1	35.4 ± 4.2	0.7624
	**reconstituted serum**	2.5%	26.7 ± 3.9	-	-
		5%	83.0 ± 10.0	-	-
		10%	106.2 ± 20.6	-	-

IL-6 (interleukin-6), HS (heparan sulfate). Data represent the mean ± SD of triplicate samples, representative of three independent experiments. P values represent statistically significance between untreated and treated cells with peptide 19–2.5 using multiple t-test with Holm-Šídák correction.

### Soluble HS in human septic shock serum

To determine the effects of soluble HS in serum from patients with septic shock, we first measured levels of soluble HS in the applied serum (Table 1). Next, we eliminated soluble HS from the serum and stimulated HL-1 cells with HS-free serum from patients with Gram-negative or Gram-positive septic shock. We found a significant lower NFκB-luciferase reporter activity compared to cells stimulated with the primary serum (2,5%, 5%, and 10%, respectively, all p < 0.05) (HS-free serum see Fig 4C and 4D, primary serum see Fig 4A and 4B). Treatment with peptide 19–2.5 did not significantly alter NFκB-luciferase reporter activity in HL-1 cells stimulated with HS-free serum compared to untreated cells (2,5%, 5%, and 10%, respectively, all n.s.) (Fig 4C and 4D). To exclude that other relevant factors had been co-eliminated during HS elimination we reconstituted the detected amount of HS using artificial HS to each serum sample and re-performed the measurements. In cells stimulated with reconstituted serum we obtained nearly the same elevated NFκB-luciferase reporter activity (Fig 4C and 4D), than in cells stimulated with primary serum (Fig 4A and 4B). Concomitantly to the assessment of NFκB-luciferase reporter activity, we measured TNF-α and IL-6 mRNA and secreted protein concentrations of cells stimulated with HS-free serum (stimulation with 5% HS-free serum see center part of Fig 5A–5H, other concentrations see Tables 2 and 3). Application of HS-free serum induced significant lower levels of both TNF-α and IL-6 mRNA and secreted protein concentrations compared to primary serum (2,5%, 5%, and 10%, respectively, all p < 0.05). Treatment with peptide 19–2.5 did not significantly alter TNF-α and IL-6 mRNA expression and secreted protein concentrations in HL-1 cells stimulated with HS-free serum compared to untreated cells (Fig 5A–5H, middle part and Tables 2 and 3). Application of reconstituted serum to HL-1 cells resulted in increased levels of both TNF-α and IL-6 mRNA and secreted protein concentrations compared to cells stimulated with HS-free serum (2,5%, 5%, and 10%, respectively, all p < 0.05), which were comparable to primary serum (Fig 5A–5H, right part and Tables 2 and 3).

### Study population characteristics

**Fig 6:**
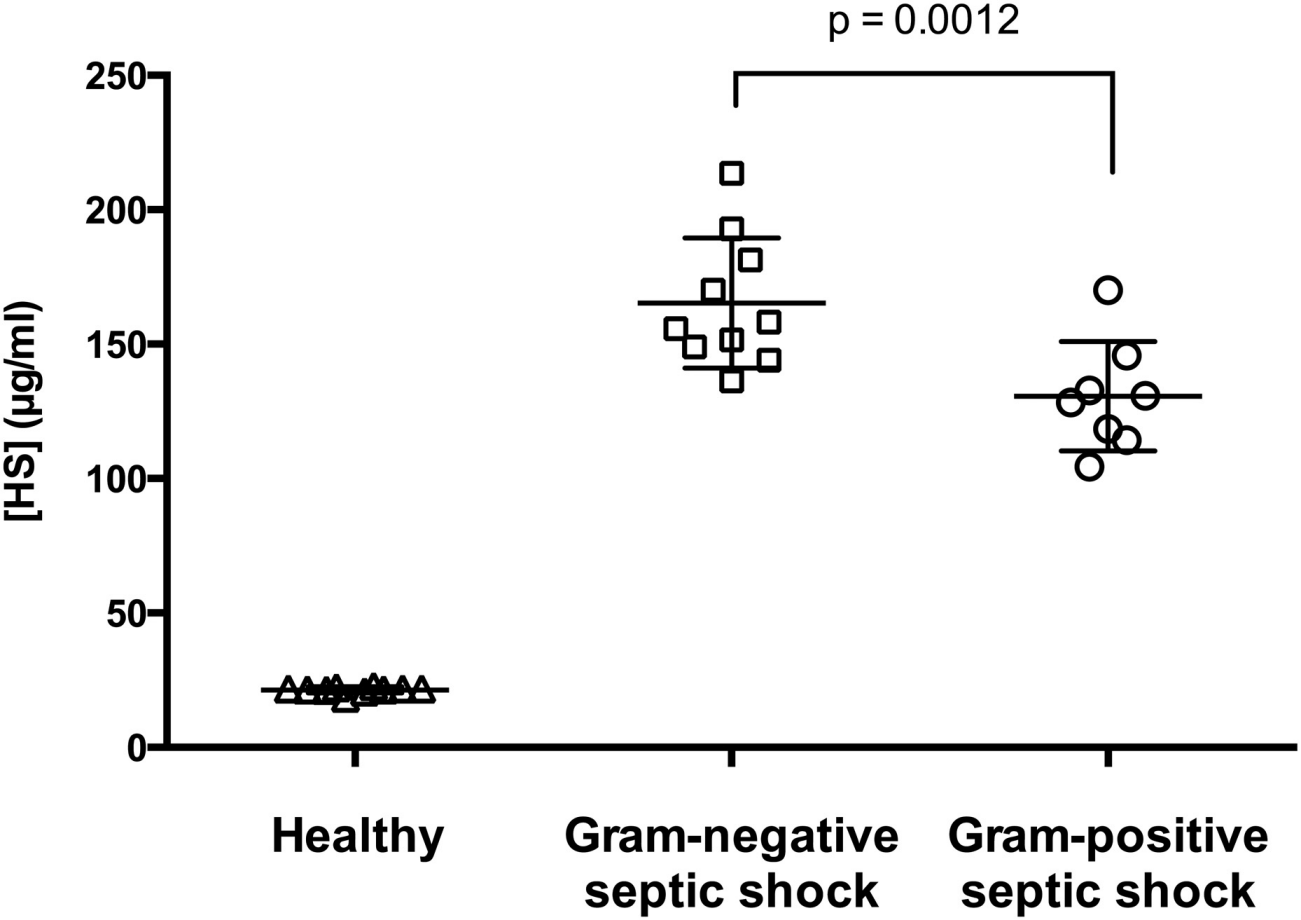
Heparan sulfate levels in human and murine sepsis. Heparan sulfate levels were measured in plasma of healthy humans (n = 10) as well as of patients with Gram-negative (n = 10) or Gram-positive (n = 8) septic shock using ELISA. Data represent the mean ± SD of duplicate samples. P value represent the statistically significance between HS level in Gram-negative and Gram-positive septic shock using 1-way-ANOVA and Tukey´s-Test for multiple comparisons.

Included patients with septic shock had a mean age of 70 ± 15 years (78% male). The healthy volunteers had a mean age of 67 ± 19 years (50% male). HS level were significantly higher in patients with septic shock compared to healthy volunteers (p < 0.0001). There was a significant difference of HS levels between the patients with Gram-negative to those with Gram-positive septic shock (Fig 6). Additional characteristics of patients’ sera used for cell stimulation (n = 6) are shown in Table 1.

## Discussion

This study demonstrates that treatment with peptide 19–2.5 decreases inflammatory response in HL-1 cells stimulated with both PAMPs and DAMPs. Furthermore our work shows that soluble HS in serum from patients with Gram-negative or Gram-positive septic shock induces a strong pro-inflammatory response in HL-1 cells, which can be effectively blocked by peptide 19–2.5.

### Peptide 19–2.5 reduces PAMP-associated inflammation

LPS and FSL-1 are highly potent immune stimulatory bacterial cell wall compounds. They are mainly known for triggering Gram-negative or Gram-positive sepsis [23]. It was shown that peptide 19–2.5 changes the aggregate structure of LPS. The lipid A part of LPS is converted from its cubic aggregate structure into an inactive multilamellar structure, thereby preventing the binding of LPS to Toll-like receptor 4 (TLR4) [15]. Biophysical studies on FSL-1:peptide 19–2.5 interaction indicate a similar mechanism, leading to FSL-1 neutralization in the biological experiment (unpublished data). As expected, we found attenuated NFκB-luciferase reporter activity as well as decreased cytokine mRNA levels and secreted protein concentrations of HL-1 cells stimulated with LPS or FSL-1 in the presence of peptide 19–2.5.

### Peptide 19–2.5 reduces DAMP-associated inflammation

Because inhibition of pro-inflammatory cytokine release through DAMPs during sepsis may provide a suitable approach to anti-infective therapy [12] we investigated the pro-inflammatory response in HL-1 cells stimulated with several concentrations of HS in the presence or absence of peptide 19–2.5. We measured a dose-dependent increase in NFκB-luciferase reporter activity as well as elevated cytokine mRNA levels and secreted protein concentrations. These results are in line with findings from Johnson et al. who stimulated different cell lines with HS concentrations from 0.3 to 10 μg/ml and detected a dose-dependent NFκB activation, notably after 30-min stimulation [10], compared to 4h stimulation in our experiments. As part of the innate immune system, Toll-like receptors rapidly react on a pathogen challenge without prior exposure. HS are known as endogenous TLR-4 ligands [9,10,24–31], which induce the release of cytokines [32] and trigger the pro-inflammatory cascades in severe sepsis and septic shock [9,10]. Our work is the first that identifies peptide 19–2.5 as a potential therapeutic option of blocking the HS-associated inflammatory response. Recently, an investigation showed that peptide 19–2.5 binds to HS moieties attached to their proteoglycan on cells, thereby inhibiting the entry of enveloped viruses [18]. Similarly to the described changes of the LPS aggregate structure by peptide 19–2.5 [15] the decreased pro-inflammatory response by peptide 19–2.5 in cells stimulated with HS could be explained by neutralization of the HS charge. It was described before that peptides interfering with protein-protein or viral protein-host membrane interfaces may have the potential to serve as novel antiviral drugs [33]. Krepstakies et al. investigated the binding of the positively charged peptide 19–2.5 to the negatively charged HS by biophysical analysis. They detected an alteration of the peptide’s secondary structure and a characteristic change in the hydration and sulfation status of the HS moieties due to a pronounced interaction of peptide 19–2.5 and HS [18]. Recently, it was shown that high sulfation in O-position of HS is required for their immunomodulatory activities [34]. Thus, reduction of pro-inflammatory response in HL-1 stimulated with soluble HS (Fig 3) may be explained by neutralization of the HS structure by peptide 19–2.5, impeding binding of HS to Toll like receptor 4.

### Soluble HS in serum from septic shock patients

Although several studies have evaluated circulating levels of glycosaminoglycans in plasma of critically ill patients [35–37], our work is the first to identify a difference in HS levels according to the type of bacterial infection (Fig 6). In addition to the pro-inflammatory response in HL-1 cells stimulated with HS, incubation with serum from septic shock patients also induced an inflammatory response (Figs 4 and 5). Our measurements are consistent with another study using sera (2.5–10%) collected from mice 4 h after sepsis induced by cecal ligation and puncture (CLP) [38]. Data from this model suggest the time-dependent generation of inflammatory cell injury in primary cultures of mouse cortical tubular epithelial cells [38].

Johnson et al. administered HS by intraperitoneal injection to mice. Eighty percent mice injected with HS died, however 5 mg of HS for intraperitoneal injection was used [9]. To determine the relevance of soluble HS in human serum for an inflammatory response, we eliminated HS from serum and found significantly attenuated inflammatory response relative to that observed after exposure to primary serum from patients with septic shock (Figs 4 and 5). Notably, addition of peptide 19–2.5 to the HS-free serum did not alter the inflammatory response, suggesting an HS-dependent mechanism of peptide 19–2.5. It has been well documented that HS binds an array of growth factors, chemokines and cytokines [39]. Indeed, there have been many cases in which factors were studied using elimination experiments, which was later found to be not reproducible due to co-elimination of other factors [40]. To exclude other than HS effects after elimination, we reconstituted the detected amount of HS to each serum sample and re-performed the measurements using artificial HS. Stimulation with reconstituted serum reproduced the increase in NFκB-luciferase reporter activity, cytokine mRNA levels and secreted protein concentrations as detected after stimulation with primary serum (Figs 4 and 5 and Tables 2 and 3).

Yet, there are some limitations of our study. First, we investigated only the early phase of sepsis in humans. The results may differ in later stages of sepsis after initial improvement by adequate therapy. Second, the use of a cell culture model to study peptide treatment limits the transferability to human sepsis. Third, although we showed that HS induces inflammatory responses in murine cardiomyocytes, our findings are limited to *in vitro* measurements. Thus we will further investigate the role of HS in triggering cardiac inflammation and dysfunction during sepsis *in vivo*.

In summary, our data indicate for the first time that the treatment with peptide 19–2.5 decreases the inflammatory response in HL-1 cells stimulated with either PAMPs or DAMPs. Moreover, we demonstrated for the first time that soluble HS in serum from patients with Gram-negative or Gram-positive septic shock induces a strong pro-inflammatory response in HL-1 cells, which can be effectively blocked by peptide 19–2.5. Thus, to our knowledge peptide 19–2.5 is the only anti-infective agent interacting with *both* PAMPs and DAMPS, suggesting peptide 19–2.5 may have the potential for further development as a broad-spectrum anti-inflammatory agent in sepsis-induced myocardial inflammation and dysfunction.
